# WC/C Composite as an Efficient Photothermal Material for Solar-Driven Seawater Evaporation

**DOI:** 10.3390/nano16120738

**Published:** 2026-06-13

**Authors:** Shixu Dong, Weifeng Li, Yumei Long

**Affiliations:** 1 College of Chemistry, Chemical Engineering and Materials Science, Soochow University, Suzhou 215123, China; 2 The Key Lab of Health Chemistry and Molecular Diagnosis of Suzhou, Soochow University, Suzhou 215123, China

**Keywords:** photothermal conversion, WC/C composite, solar-driven interfacial water evaporation, seawater evaporation

## Abstract

Solar-driven interfacial water evaporation has been recognized as an effective measure to address freshwater scarcity. Photothermal materials lie at the core of this process and have been extensively studied. However, conventional carbon-based materials typically suffer from high thermal emissivity, leading to significant heat loss. Here, we report a tungsten carbide/carbon composite polyvinyl alcohol hydrogel evaporator (PWC) for solar-driven interfacial seawater evaporation. Specifically, a tungsten carbide/carbon (WC/C) composite was synthesized via a straightforward one-step molten salt coating method and exhibited a remarkable photothermal conversion efficiency of 67.1%, attributed to the plasmon resonance absorption effect of WC nanoparticles. When incorporated into a polyvinyl alcohol (PVA) hydrogel via a physical-chemical dual-crosslinking strategy, the resulting PWC evaporator achieved a high evaporation rate of 2.99 kg m^−2^ h^−1^ and a conversion efficiency of 90.9% in a 5 wt% NaCl solution under 1 kW m^−2^ illumination. In addition, the evaporator can purify seawater and effectively remove a variety of organic dyes. This study provides a viable strategy for a sustainable freshwater supply.

## 1. Introduction

With the continuous growth of the global population and industrialization, freshwater resources have become scarce [1]. Hence, a variety of water treatment technologies have been developed to produce fresh water, including reverse osmosis and multi-stage distillation. Nevertheless, these technologies face significant barriers to practical implementation, such as high energy consumption and complex operational requirements [2,3]. In comparison, solar-driven interfacial evaporation (SDIE) based on photothermal materials has emerged as a sustainable, cost-effective, and environmentally friendly technology for freshwater production, holding the potential to alleviate water scarcity and pollution [4].

In the past few years, the SDIE system has utilized photothermal materials to convert absorbed solar energy into heat and confine it to the air–water interface, reducing heat loss and enabling high-efficiency steam generation [5,6]. A key functional component of solar-driven interfacial evaporators is photothermal materials, which significantly influence the overall evaporation efficiency [7]. At present, common photothermal materials mainly include plasmonic materials, carbon-based materials, semiconductor materials, and polymeric materials [8,9]. Among these photothermal materials, carbon-based materials, such as carbon nanotubes [10], carbon black [11], carbon dots [12], and graphene [13], have been widely employed in solar-driven water evaporation systems due to their strong broadband light absorption and excellent thermal stability [14,15]. However, carbon-based materials suffer from high emissivity due to surface reflection [16,17,18], which limits their photothermal conversion efficiency. To date, multiple approaches have been developed to address this issue. The design of composite materials enhances solar response through the synergistic effect between their components [19]. Specifically, the combination of plasmonic metals and carbon-based materials has been reported to further enhance photothermal performance by harnessing plasmonic effects [20,21]. For example, Wang et al. [22] successfully constructed a biomass-derived aerogel framework modified with copper nanoparticle-decorated carbon nanotubes (Cu@CNTs-IP). The strong light absorption of the carbon nanotubes, combined with the localized surface plasmon resonance (LSPR) effect of copper, achieved an evaporation rate of 1.28 kg m^−2^ h^−1^ under 1 sun illumination. Plasmonic metal materials, especially Au and Ag, are extensively employed in studies of solar interfacial evaporation [23,24]. Su et al. [25] reported the modification of partially reduced graphene oxide nanosheets with Au nanorods. The synergistic effect between graphene nanosheets and gold nanorods demonstrated a high level of light responsiveness. Nonetheless, the widespread application of precious metals is limited by their high cost and inadequate chemical stability in practical applications [26,27]. In comparison, transition metal carbides (TMCs) exhibit comparable plasmonic resonance effects to those of precious metals while demonstrating superior performance in terms of synthesis conditions, environmental compatibility, and solar absorption efficiency [28,29]. For instance, Jing et al. reported that a composite of CQDs and Ti_3_C_2_TX served as a photothermal material, exhibiting high light absorption and photothermal conversion efficiency for evaporator fabrication [30]. Furthermore, tungsten carbide (WC) has been reported as a promising photothermal material candidate that exhibits high chemical stability [31] and distinct LSPR effects [32,33], leading to strong absorption across the entire solar spectrum from 200 nm to 2500 nm. Han et al. [32] achieved a solar-to-vapor efficiency of 90.8% by integrating plasmonic WC nanomaterials into a structural design featuring a bottom layer with discrete hydrophobic and hydrophilic regions. Consequently, nanostructured tungsten carbide has emerged as an ideal candidate for photothermal conversion in solar-driven desalination.

Herein, we demonstrate a facile method for synthesizing WC nanoparticles supported on carbon nanosheets via a molten salt coating process under an air atmosphere. Meanwhile, the plasmon resonance effect and interband transitions of WC enhance the photothermal conversion efficiency of the carbon-based materials, contributing to a 97% absorption rate for the WC/C composite. Subsequently, the composite was incorporated into a PVA hydrogel matrix to fabricate an evaporator with a crosslinked network by physicochemical crosslinking. Consequently, the prepared PWC hydrogel evaporator achieved an evaporation rate of 2.99 kg m^−2^ h^−1^ and an evaporation efficiency of 90.9% in a 5 wt% NaCl solution under 1 kW m^−2^ illumination. Furthermore, the PWC hydrogel exhibited excellent salt resistance and sewage purification capability, positioning it as an effective photothermal conversion material for seawater desalination.

## 2. Materials and Methods

### 2.1. Materials

Ammonium metatungstate ((NH_4_)_6_H_2_W_12_O_40_·xH_2_O, AMT) was purchased from Sinopharm Chemical Reagent Co., Ltd. (Shanghai, China). Dicyandiamide (C_2_H_4_N_4_, DCD), glutaraldehyde (GA, 50% aqueous solution), and polyvinyl alcohol (PVA-124, Mw: 105,000) were obtained from Macklin Biochemical Technology Co., Ltd. (Shanghai, China). Sodium dodecyl sulfate (SDS) was obtained from Solarbio Science and Technology Co., Ltd. (Beijing, China). Sodium chloride (NaCl) and potassium bromide (KBr) were acquired from Aladdin Reagent Co., Ltd. (Shanghai, China). Hydrochloric acid (HCl, 37%) was sourced from Ling Feng Chemical Reagent Co., Ltd. (Shanghai, China). All reagents were used as received without further purification.

### 2.2. Preparation of WC/C Composite

The synthesis procedure began by uniformly mixing 0.1 g of ammonium metatungstate (AMT) and 1 g of dicyandiamide (DCD) in an agate mortar, followed by the addition of 1.1 g of sodium chloride (NaCl) with continued grinding until complete homogenization. A predetermined amount of potassium bromide (KBr) was then employed to encapsulate the (DCD/AMT/NaCl) powder mixture via the KBr salt-matrix method, which was subsequently compacted into pellets through cold isostatic pressing (designated as DCD/AMT/NaCl@KBr). The pellets were thermally treated with a controlled heating rate of 5 °C min^−1^ up to 900 °C under an air atmosphere, maintained at this temperature for 2 h, and then allowed to cool to room temperature. The resulting products were subjected to multiple washing cycles with deionized water and finally dried overnight at 60 °C. By systematically varying the DCD/AMT mass ratios, five composite materials were successfully synthesized, labeled as DCD/AMT-2, DCD/AMT-4, DCD/AMT-6, DCD/AMT-8, and DCD/AMT-10, corresponding to mass ratios of 0.2 g DCD:0.1 g AMT, 0.4 g DCD:0.1 g AMT, 0.6 g DCD:0.1 g AMT, 0.8 g DCD:0.1 g AMT and 1.0 g DCD:0.1 g AMT, respectively.

### 2.3. Preparation of PWC Hydrogel

The synthesis was initiated by dissolving 1.2 g of PVA in 10.8 mL of deionized water at 90 °C to form a 10 wt% aqueous solution. Subsequently, varying amounts of the WC/C composite (0.025 g, 0.05 g, 0.075 g, 0.10 g, and 0.15 g) were incorporated into the PVA solution under vigorous stirring. Sodium dodecyl sulfate (SDS) was then introduced to the mixture followed by mechanical agitation, after which 75 μL of glutaraldehyde (GA) and 200 μL of hydrochloric acid were added to induce gelation for 3 h. Then, the hydrogels were frozen at −20 °C for 12 h and thawed at room temperature for four cycles. To remove unreacted crosslinking agents, the hydrogel was immersed in deionized water for solvent exchange [34]. The prepared hydrogels containing different WC/C loadings were systematically designated as PWC-25, PWC-50, PWC-75, PWC-100, and PWC-150, where the numerical suffixes represent the added WC/C mass in milligrams.

### 2.4. Characterization

The morphological and microstructural characteristics of the samples were characterized by scanning electron microscopy (SEM, SU8010, Hitachi, Japan) and transmission electron microscopy (TEM, HT7700, Hitachi, Japan), while the material structure was analyzed using X-ray diffraction (XRD, D8 Advance, Bruker, Germany), Raman spectroscopy (Raman, LabRAM Soleil, HORIBA, Japan), and Fourier transform infrared spectroscopy (FTIR, VERTEX 70+HYPERION 2000, Bruker, Germany). The reflectance and absorbance spectra in the 300–2500 nm wavelength range were measured by ultraviolet–visible–near infrared spectrophotometry (UV–Vis–NIR, UV3600, Shimadzu, Japan). Elemental composition was determined through energy-dispersive spectroscopy (EDS) and X-ray photoelectron spectroscopy (XPS, EXCALAB 250 XI, Thermo Scientific, USA). Thermal stability under a nitrogen atmosphere in the temperature range of 30–200 °C was evaluated by differential scanning calorimetry (DSC, NETZSCH STA 499C, Netzsch, Germany). The rheological properties of the hydrogels were measured using a rotational rheometer (RR, Haake RS6000, Thermo Fisher Scientific, Germany). The concentrations of the main ions before and after desalination were measured by inductively coupled plasma optical emission spectroscopy (ICP-OES, Agilent 5110, Agilent Technologies, Santa Clara, CA, USA).

### 2.5. Solar Driven Interfacial Water Evaporation Performance Experiments

The experimental setup employed a xenon lamp equipped with an AM 1.5G filter (CEL-S500, Beijing Zhong jiao Jin yuan Technology Co., Ltd., Beijing, China) as the light source, with solar irradiance measured by an optical power meter (CEL-NP2000-2A, Beijing Zhong jiao Jin yuan Technology Co., Ltd.). Mass changes were recorded at 5-min intervals using an electronic balance (TE124S, Sartorius Scientific Instruments Co., Ltd. Germany, accuracy: 0.01 g). The PWC evaporator was secured using EVA foam and positioned in a beaker, maintaining direct contact between its bottom surface and the water interface. Real-time temperature monitoring of the evaporator surface was conducted using an infrared thermal imaging camera.

## 3. Results and Discussion

### 3.1. Preparation and Characterization of WC/C Composite

**Figure 1:**
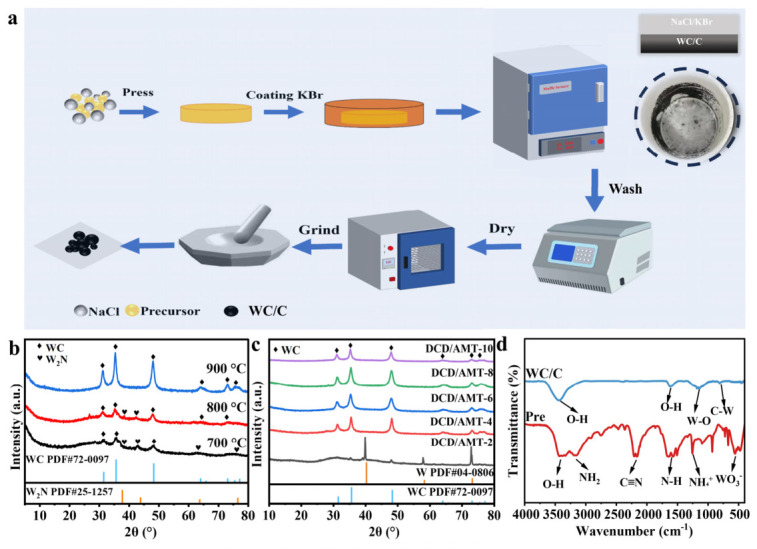
(**a**) Schematic diagram of WC/C preparation by molten salt coating method; (**b**) XRD patterns at different calcination temperatures with a DCD/AMT mass ratio of 10; (**c**) XRD patterns under 900 °C calcination with different DCD/AMT mass ratios; (**d**) FTIR spectra of WC/C and its precursor.

A one-step molten salt coating method was used to synthesize the WC/C composite material in an air atmosphere, as shown in Figure 1a. As the temperature rose, the molten salt formed a liquid coating layer that effectively isolates the sample from air contact, thereby significantly suppressing sample oxidation during the process. XRD patterns of the samples were obtained at different calcination temperatures with a DCD/AMT mass ratio of 10 (Figure 1b). At the lowest calcination temperature of 700 °C, the sample consisted of a W_2_N and WC mixed phase. The four well-defined diffraction peaks at 37.7°, 43.8°, 63.7°, and 76.5° corresponded to the (111), (200), (220), and (311) crystal planes of W_2_N (JCPDS No. 25-1257), respectively. Meanwhile, the other diffraction peaks at 31.5°, 35.6°, 48.3°, and 64.0°, which corresponded to the (001), (100), (101), and (110) crystal planes of hexagonal WC (JCPDS No. 72-0097), respectively [33,35]. When the calcination temperature increased from 800 °C to 900 °C, all characteristic peaks of W_2_N disappeared, and the sample solely exhibited the characteristic peak of hexagonal WC. Furthermore, the XRD patterns of the WC/C composite showed no diffraction peaks of carbon, as the profound crystallization of WC overshadows the weak signal from amorphous carbon. The XRD spectra of samples prepared by different mass ratios of DCD to AMT at a calcination temperature of 900 °C are shown in Figure 1c. When the DCD to AMT mass ratio was 2, the resulting sample was found to consist of metallic tungsten (JCPDS No. 04-0806) and hexagonal WC [36]. Meanwhile, when the DCD to AMT mass ratios increased from 4 to 10, the disappearance of the metallic tungsten diffraction peak indicates that a mass ratio of 4 is the lowest DCD to AMT mass ratio for synthesizing WC/C. Raman spectroscopy was employed to investigate defect structures of the resulting carbon materials. The Raman spectra of WC/C exhibited both D and G peaks, corresponding to lattice defects and the vibration of sp^2^ hybridized carbon atoms in the material [37]. Furthermore, the I_D_/I_G_ ratio confirms the presence of structural defects, indicating that the carbon is predominantly composed of defective nanosheets. Carbon nanosheets exhibit broadband light absorption owing to their sp^2^-conjugated structure. Meanwhile, the structural defects function as non-radiative recombination centers, promoting the non-radiative recombination of photogenerated carriers and thereby achieving high-efficiency photothermal conversion. The value of I_D_/I_G_ increased from 0.91 to 0.98 as the DCD to AMT mass ratio used in the synthesis of WC/C was raised from 2 to 10 (Figure S1), indicating that a higher ratio of DCD to AMT contains more defective carbon. The increased defective carbon content in the WC/C composite provides ample anchoring sites for the uniform dispersion of WC nanoparticles while simultaneously enhancing light absorption across the entire solar spectrum, thereby boosting photothermal conversion efficiency. The resulting FTIR spectrum showed that there were significant differences in the structure between the precursor and the WC/C composite (Figure 1d). The precursor exhibited characteristic peaks at 3400 cm^−1^ and 3230 cm^−1^, corresponding to the stretching vibration peaks of –OH and –NH_2_ [38]. Notably, the peaks at 2191 cm^−1^ and 1640 cm^−1^ were respectively attributed to the stretching vibration of C≡N in DCD and the bending vibration of N–H (Figure 1d) [39]. The disappearance of the cyano (–CN) and amino (–NH_2_) functional groups provided further evidence that DCD had undergone high-temperature thermal decomposition. Additionally, the broad peak at 400–1000 cm^−1^ that corresponded to the W-O stretching vibration disappeared after pyrolysis and was replaced by the absorption peak of the W-C bond. This result indicates that ammonium metatungstate undergoes pyrolysis to form the intermediate phase tungsten oxide, which is thermally reduced to WC in the presence of a carbon source. As shown in Figure S2a, the XPS spectrum confirms that the WC/C composite consisted of elements of C, W, and O. The O 1s XPS spectrum exhibited peaks at 530.8, 531.7, and 533.3 eV, which were attributed to W–O, C–O, and adsorbed water, respectively, confirming that the WC/C composite forms an oxide layer when exposed to air (Figure S2b) [40]. The XPS spectrum of W 4f (Figure S2c) presented multiple peaks, indicating that W exists in chemical states in the sample. The peaks centered at 32.5 eV (W 4f_7/2_) and 34.6 eV (W 4f_5/2_) corresponded to W^4+^, indicating the presence of WC. The other two peaks, centered at 35.9 eV and 38.0 eV, can be attributed to W^6+^, which arises from the oxidation of exposed WC on the surface to WO_3_ when exposed to air [41]. Meanwhile, the C 1s XPS spectrum in Figure S2d showed three peaks at 284.1 eV, 284.8 eV, and 285.8 eV, corresponding to C-W, C-C, and C-O, respectively. This further confirms the successful synthesis of the WC/C composite [42].

### 3.2. Morphological Characterization of WC/C Composite

**Figure 2:**
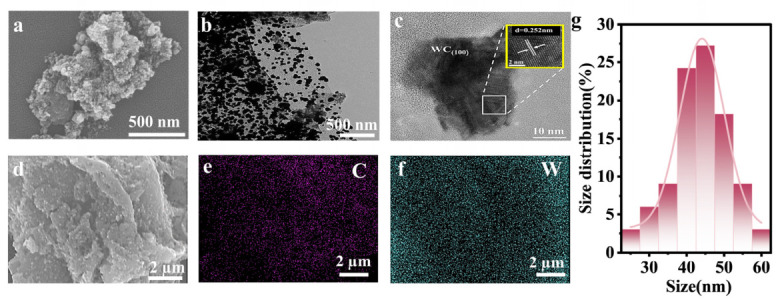
(**a**) SEM image of WC/C; (**b**) HRTEM image of WC/C; (**c**) HRTEM image of WC/C; (**d**–**f**) elemental mapping distribution of WC/C; (**g**) particle size distribution of WC/C.

Figure 2a presents a scanning electron microscopy (SEM) image of the WC/C composite, revealing that WC is present as nanoparticles dispersed on the surface of carbon nanosheets. As illustrated in Figure 2b, TEM revealed that WC nanoparticles were uniformly dispersed on the carbon nanosheet surface. This uniform dispersion is attributed to the high surface area provided by the nanosheets. In addition, more loading sites for WC were achieved by increasing the DCD to AMT mass ratio, which tunes the relative proportions of WC and carbon nanosheets (Figure S3). The HRTEM image in Figure 2c shows that the nanoparticles had an irregular shape. The spacing between two lattice fringes was 0.25 nm, corresponding to the (100) crystal plane spacing of hexagonal WC [33,43]. Elemental mapping (Figure 2d–f) clearly demonstrated the homogeneous distribution of W and C atoms, indicating that WC was uniformly dispersed on the carbon nanosheets. The mass percentages of C and W were 35.93% and 64.07%, respectively (Figure S4). Based on these data, the WC-to-carbon mass ratio in the sample was calculated to be 2.15:1 (see Note S1 and Table S1). Furthermore, Figure 2g shows that the particle size of the WC/C composite was concentrated in the range of 40–50 nm, which is significantly smaller than that of the carbon nanosheets. This substantial size disparity facilitates intimate and uniform contact between WC nanoparticles and the carbon nanosheet surface.

Further insights into the formation mechanism of the sample were gained by TG-DTA analysis of the precursor under a N_2_ atmosphere (Figure S5). The initial mass loss of 3.96%, which coincided with an endothermic peak in the DTA curve, corresponded to the removal of adsorbed water from the precursor. Furthermore, in the temperature range of 210 °C to 327 °C, a mass loss of 37.24% was observed. Correspondingly, the DTA curve exhibited a prominent peak within the same range, indicating the thermal decomposition of DCD and AMT, which leads to the formation of WO_3_ and the release of HCN. When the temperature reached 592 °C, the weight dropped dramatically by 50.51%. This loss is attributed to the carbothermal reduction reaction of tungsten oxide by graphitic carbon nitride derived from the precursor, during which tungsten oxide is reduced to metallic W and WC. Meanwhile, the dynamic molten salt (KBr/NaCl) environment facilitates the dispersion of WC onto carbon nanosheets derived from the high-temperature decomposition of dicyandiamide. Thus, the above results demonstrate the successful preparation of the WC/C composite.

### 3.3. Photothermal Performance of WC/C Composite

**Figure 3:**
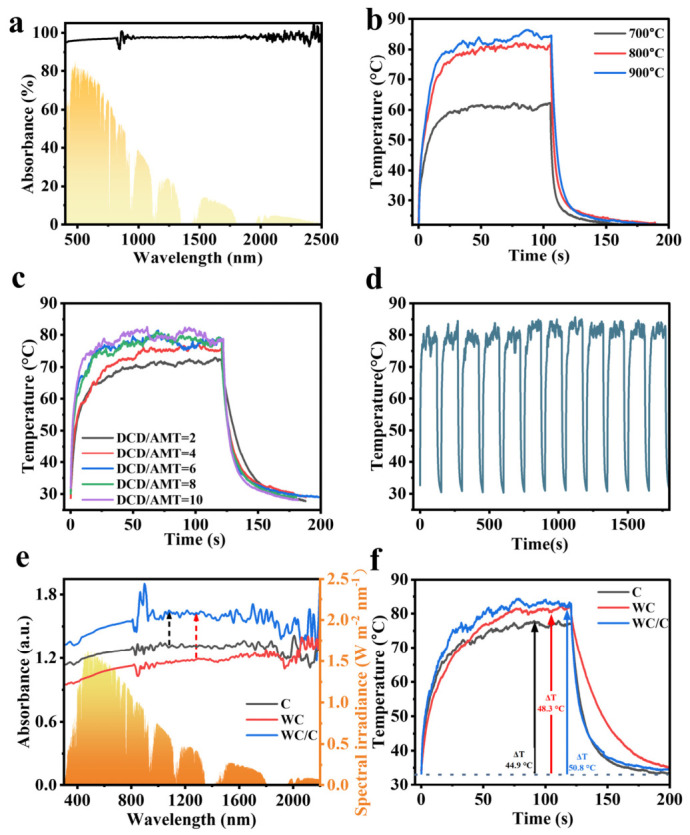
(**a**) Absorption spectra of the WC/C composite. (**b**) Photothermal properties of the WC/C composite synthesized at different calcination temperatures. (**c**) Photothermal properties of WC/C prepared with different DCD/AMT mass ratios. (**d**) Photothermal cycling properties of the WC/C composite. (**e**) Light absorption of the WC/C composite in contrast with WC and C. (**f**) Photothermal curves of WC/C, WC, and C under 1 sun illumination.

The sunlight absorption capacity of samples is very important for efficient water evaporation. Accordingly, the light absorption capacity of the WC/C composite was measured by a UV–Vis–NIR spectrophotometer. As shown in Figure 3a, the absorption rate of the full spectrum was 97%. The photothermal properties of material were characterized using an infrared (IR) camera under 1 kW m^−2^ illumination. As shown in Figure 3b, the temperature of the WC/C composite calcined at 900 °C increased rapidly from room temperature to 80 °C during 25 s, indicating its optimal photothermal performance.

**Figure 4:**
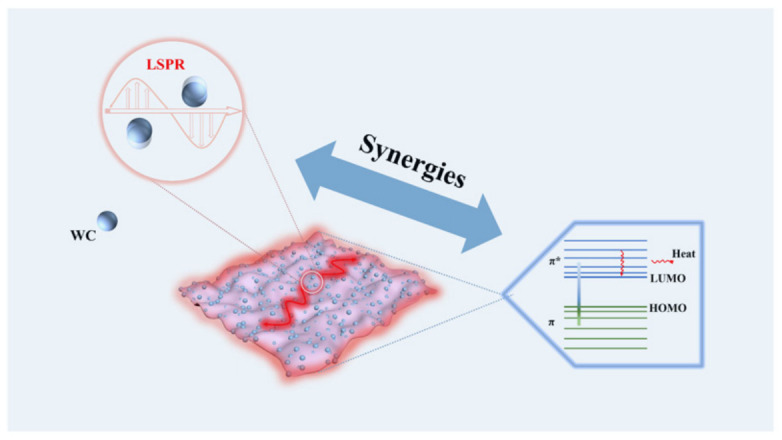
Photothermal conversion mechanism schematic diagram of carbon and WC NPs.

Figure 3c displays the photothermal performance of samples calcined at 900 °C with different DCD to AMT mass ratios. As the DCD to AMT mass ratio increased from 2 to 10, the surface temperature of the samples rose from 72.1 °C to 82.1 °C, consistent with the progressive increase in light absorption intensity (Figure S6). Furthermore, when the mass ratio of DCD to AMT was 10, the samples exhibited a rapid thermal response due to the synergistic interaction between WC and carbon materials. Similarly, the sample retained a temperature of 80 °C after undergoing 12 cycles (Figure 3d), further substantiating the excellent thermal stability of the material. Simultaneously, the temperature increments (ΔT) of all samples exhibited a power density-dependent photothermal effect under various laser power densities (Figure S7a,b), indicating that the photoresponse of WC/C is affected by the light power density. In summary, the WC/C composite with a DCD to AMT mass ratio of 10 calcined at 900 °C was selected as the research object for subsequent studies. This is because the WC nanoparticles are supported by abundant carbon nanosheets, which facilitate heat transfer within WC through the carbon nanosheets, thereby achieving broadband absorption capability. The photothermal conversion efficiency of photothermal materials severely restricts the performance of solar evaporators. Therefore, the optical absorption capacities of WC, C, and WC/C were investigated by UV–Vis–NIR spectroscopy, as shown in Figure 3e. The light absorption performance of WC/C across the broadband solar spectrum was higher than that of WC and pure carbon, indicating that the synergistic effect between WC and carbon enhances its light absorption ability beyond that of single photothermal materials. Similarly, Figure 3f shows that the temperature of the WC/C composite exhibits a much faster heating rate and ultimately reaches a higher temperature under 1 kW m^−2^ illumination. The temperature of the WC/C composite increased by 50.8 °C above room temperature, which was 5.9 °C higher than that of carbon and 2.5 °C higher than that of WC. From the cooling curves (Figures S8–S10, Note S2, and Table S2), the photothermal conversion efficiency of WC/C reached 67.1%, significantly higher than those of pure carbon (44.6%) and WC (51.7%). These results further confirm that the synergistic effect of carbon and WC enables the efficient absorption of solar energy and its conversion into thermal energy. This outstanding photothermal performance can be attributed to the following mechanism. The photothermal conversion mechanism of the WC/C composite arises from the synergistic interplay of two effects: the localized surface plasmon resonance (LSPR) of WC nanoparticles and the molecular thermal vibration of the carbon-based materials. The LSPR effect of WC NPs enables efficient sunlight harvesting at specific resonance wavelengths, generating local hotspots and a rapid thermal response. Meanwhile, the π bonds of carbon enable broadband absorption, extending the absorption spectrum into the infrared band (Figure 4). This synergy underpins the outstanding photothermal performance of the WC/C composite.

### 3.4. The Preparation and Structural Characterization of PWC

**Figure 5:**
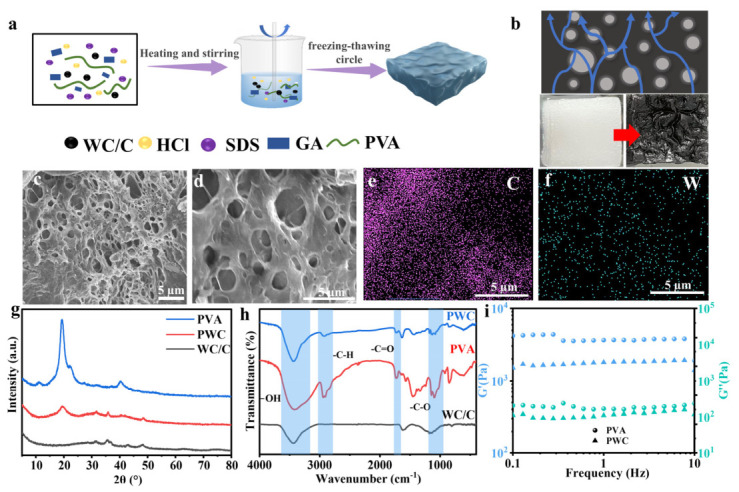
(**a**) Schematic diagram of the PWC hydrogel evaporator preparation. (**b**) Pore structure of the PWC hydrogel and digital photos of as-prepared PVA hydrogel and PWC. (**c**) SEM image of the PWC hydrogel. (**d**–**f**) Elemental mapping distribution of WC/C. (**g**) XRD patterns of PVA, PWC, and WC/C. (**h**) FTIR spectra of PWC, PVA, and WC/C. (**i**) Rheological curves of the PVA hydrogel and PWC.

The preparation process of the WC/C composite hydrogel (PWC) is shown in Figure 5a. The hydrogel was fabricated through chemical crosslinking and physical freeze–thaw cycles. Figure 5b demonstrates that the interconnected pore network provides an efficient channel for water transport to the photothermal surface. Furthermore, the digital photo of the pure PVA hydrogel appeared white, whereas the PWC hydrogel appeared black upon incorporation of the WC/C composite. The SEM image (Figure 5c) reveals that the PWC hydrogel possesses an abundant multi-level pore structure, providing efficient pathways for water transport and thus ensuring a continuous and stable water supply during evaporation. Notably, this unique structural design allows incident light to refract within the channels, significantly enhancing light capture and utilization efficiency [44].

The coverage of WC/C on the hydrogel framework was further confirmed through SEM-EDS analysis, revealing the distribution of W and C on the PVA skeleton surface (Figure 5d–f). In addition, the loading state of the WC/C composite in the PWC was investigated by XRD analysis, as illustrated in Figure 5g. The characteristic diffraction peaks observed at 31.5°, 35.6°, and 48.3° correspond to the (001), (100), and (101) crystal planes of the WC/C phase in the WC/C composite [33], indicating the successful loading of WC/C onto the PWC hydrogel. Simultaneously, the XRD pattern showed a diffraction peak at 19.1° corresponding to PVA, the intensity of which was significantly diminished after WC/C incorporation into the PWC hydrogel [45]. This result further confirms the successful incorporation of WC/C nanoparticles into the PVA framework. The FTIR spectra of the WC/C, PVA, and PWC hydrogels are presented in Figure 5h. The characteristic peaks of the PVA hydrogel were similar to those of the PWC hydrogel, indicating that WC/C incorporation does not alter the PVA hydrogel framework. The broad absorption band at 3300–3400 cm^−1^ is attributed to the stretching vibration of -OH in the PVA hydrogel. The characteristic peak at 2900 cm^−1^ corresponds to the stretching vibration of -C-H, and the peaks at 1723 and 1043 cm^−1^ correspond to the stretching vibration of C═O and C–O [46]. However, the peak intensity of –OH vibration in PWC hydrogel markedly decreased due to the incorporation of WC/C, indicating that functional group interactions influence the PVA hydrogel. Furthermore, the viscoelastic properties of hydrogels can be systematically characterized by the storage modulus (G′) and loss modulus (G″), where G′ reflects the elastic energy storage capacity of the material and G″ represents its viscous dissipation characteristics [47]. As illustrated in Figure 5i, both PVA and PWC hydrogels exhibited G′ values higher than their respective G″ values, confirming the formation of stable three-dimensional crosslinked networks. The lower G′ and G″ values of the PWC hydrogel reflect the restriction of polymer chain relative sliding upon the addition of WC/C nanoparticles [48]. These results indicate that WC/C nanoparticles were successfully incorporated into the PVA framework.

### 3.5. Characterization of Photothermal Water Evaporation Performance by PWC

**Figure 6:**
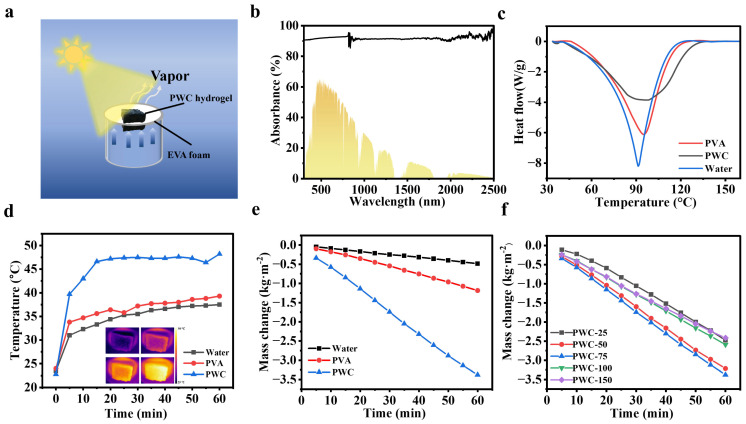
(**a**) Schematic diagram of the solar interfacial water evaporation device. (**b**) The absorption spectra of PWC. (**c**) Heat flow–temperature curves for pure water, PVA, and PWC (DSC). (**d**) Surface temperature variations of pure water, PVA, and PWC under 1 sun illumination. (**e**) Mass changes the evaporation rates of pure water, PVA, and PWC with different DCD/AMT mass ratios under 1 sun illumination. (**f**) Mass change during evaporation of PWC with different WC/C contents under one sun illumination.

The schematic diagram of the solar interfacial water evaporation system is illustrated in Figure 6a, where the top of the PWC hydrogel acts as a photothermal conversion layer to directly absorb solar radiation, while the bottom is in contact with water to achieve efficient water transfer. EVA foam, on the other hand, serves as both an insulating layer and a structural support, ensuring the stability of the evaporator and the thermal localization effect. As shown in Figure 6b, the PWC hydrogel demonstrated an exceptional light absorption of 90% throughout the full spectrum (wavelength range of 400–2500 nm). Furthermore, the equivalent evaporation enthalpies of pure water, PVA, and PWC were evaluated by differential scanning calorimetry (DSC) analysis (Figure 6c). The curve peaks of the PVA and PWC hydrogels exhibited significantly broad peak, indicating that their evaporation behavior differed from that of pure water. The evaporation enthalpies of pure water, PVA, and PWC were calculated to be 2284, 1828, and 1765 J g^−1^, respectively (Figure S11). Compared to pure water, PVA and PWC exhibited a significantly lower enthalpy of evaporation. During the evaporation process, water in the hydrogel existed in three forms: bound water (BW), intermediate water (IW), and free water (FW). Because IW exhibits weaker interactions with the polymer matrix and adjacent water molecules, it requires less energy for evaporation [49]. Consequently, the abundant hydrophilic functional groups of the hydrogel further enhance the proportion of activated IW, significantly improving the water evaporation rate [50,51]. The evaporation performance was evaluated by comparing the mass change of water, PVA, and PWC under 1 kW m^−2^ illumination. Real-time infrared thermal imaging revealed that the surface temperature of the PWC hydrogel increased dramatically from 24 °C to 47 °C, which was significantly higher than that of pure water (Figure 6d), with most of the heat localized on the evaporation surface. These results fully demonstrate that the PWC evaporator possesses outstanding photothermal conversion performance, which is critical for realizing thermal positioning and mitigating heat loss. Meanwhile, the mass change of bulk water under 1 kW m^−2^ illumination was investigated over 60 min (Figure 6e and Figure S12). Results indicate that pure water and the PVA hydrogel achieved evaporation rates of 0.48 kg m^−2^ h^−1^ and 1.18 kg m^−2^ h^−1^, respectively. Notably, the PWC hydrogel exhibited a markedly higher evaporation rate of 3.38 kg m^−2^ h^−1^, surpassing those of both pure water and the PVA hydrogel. Furthermore, the effect of WC/C contents on the evaporation performance of the PWC hydrogel was studied. As shown in Figure 6f, the mass loss of the PWC hydrogel initially increased and then decreased from PWC-25 to PWC-150. The corresponding evaporation rates were 2.48, 3.22, 3.38, 2.59, and 2.42 kg m^−2^ h^−1^ for WC/C contents of 25, 50, 75, 100, and 150 mg, respectively. This tendency may be ascribed to pore blockage within the PVA hydrogel as the WC/C content increases, which obstructs the transport pathways for water to reach the evaporator surface and thereby reduces the mass change of water.

### 3.6. The Salt Resistance and Mechanical Properties of PWC

**Figure 7:**
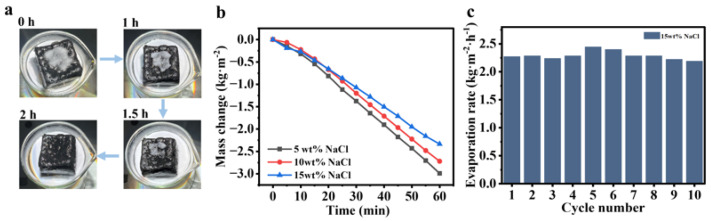
(**a**) Photos showing the salt resistance of the PWC hydrogels. (**b**) Mass change of water in NaCl solutions with different concentrations. (**c**) Evaporation rate variation of the PWC evaporator over ten cycles under one-sun illumination (15 wt% NaCl solutions).

Notably, the accumulation of salt has long been considered a significant factor hindering solar evaporators for seawater desalination. To investigate the salt resistance of the PWC evaporator, we added 1 g of NaCl to its top and captured changes on the evaporator surface. As depicted in Figure 7a, the NaCl gradually dissolved and disappeared within 2 h, indicating that the PWC evaporator has good salt resistance. This phenomenon occurs due to the gradient difference in salt concentration, which facilitates the movement of water from regions of lower salinity to regions of higher salinity [52]. To further confirm the self-cleaning performance of the PWC evaporator, an additional 1 g of NaCl was placed on the surface of the PWC hydrogel in dark conditions. After 1.5 h, the salt crystals on the PWC evaporator surface had completely disappeared (Figure S13). This rapid self-cleaning property ensures that the evaporator operates stably over the long-term. Additionally, the salt tolerance of the evaporator was tested in various salt concentrations (5 wt%, 10 wt%, and 15 wt%). As shown in Figure 7b, the PWC evaporator exhibited evaporation rates of 2.99, 2.72, and 2.34 kg m^−2^ h^−1^ under the different brine solutions, respectively. The evaporation rate of the PWC decreased as the salt concentration increased from 5 wt% to 15 wt%, which can be attributed to the increase in salt ions, which restrict the movement of water molecules and lower the saturated vapor pressure of water, consequently reducing the evaporation rate [53,54,55]. It is worth noting that the PWC hydrogel exhibited a high evaporation efficiency of 90.9% in a 5 wt% salt solution (Note S3). The long-term stability of the PWC evaporator was evaluated in simulated seawater under 1 kW m^−2^ illumination for 10 consecutive cycles. As shown in Figure 7c, the evaporation rate in 15 wt% brine remained relatively stable and an average rate close to 2.29 kg m^−2^ h^−1^, demonstrating excellent salt tolerance and durability. To confirm the stability of the PWC evaporator, a 12 h continuous test was performed, and the evaporation rate showed no obvious fluctuation (Figure S14). Furthermore, no significant change was observed on the evaporator surface after testing (Figure S15). Additionally, the PWC exhibited no severe deformation after compression by a 200 g weight and regained its original shape within a few seconds (Figure S16), indicating its excellent mechanical stability. These results indicate that the structural stability of the PWC enables its potential application under consecutive operational cycles. Moreover, compared to similar evaporators and other evaporation systems, the PWC evaporator demonstrated superior evaporation performance and efficiency (Table S3).

### 3.7. Outdoor Performance, Sewage Purification, and Seawater Desalination Capabilities of PWC

**Figure 8:**
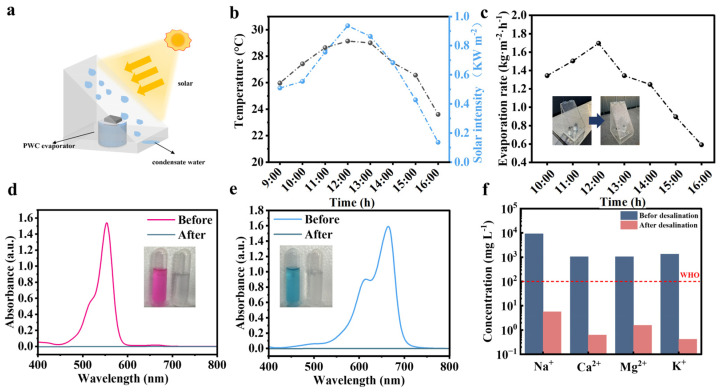
(**a**) Schematic diagram of the outdoor solar-driven seawater evaporation device. (**b**) Outdoor solar radiation intensity, ambient surface temperature variations, and (**c**) evaporation rate of the PWC evaporator during 7 h outdoor sunlight exposure on 1 December. (**d**) UV–Vis absorption spectra of RhB before and after purification. (**e**) UV–Vis absorption spectra of MB before and after purification. (**f**) Concentration of four ions (Na^+^, Ca^2+^, Mg^2+^, and K^+^) in real seawater before and after solar desalination.

To evaluate the practical outdoor application of the PWC evaporator, a simplified outdoor evaporation system was designed to test the environmental adaptability of the evaporator (Figure 8a), where water vapor condenses into droplets upon contacting the inclined inner top surface. Measurements were conducted from 9:00 to 16:00, during which surface temperature and solar intensity were recorded every hour. During the 7 h of light, solar intensity peaked at noon (12:00), corresponding to a maximum surface temperature of 30.6 °C and an evaporation rate of 1.7 kg m^−2^ h^−1^ (Figure 8b,c). After 15:00, the evaporation rate decreased correspondingly with the significant reduction in solar intensity. Moreover, to evaluate the water purification capability of the PWC hydrogel, rhodamine B (RhB, 10 mg L^−1^) and methylene blue (MB, 10 mg L^−1^) were used as simulated organic pollutants in industrial wastewater. As shown in Figure 8d,e, the UV absorption peaks of both dyes in the condensate were near zero, and the collected condensate water became clear and transparent after evaporation. This result demonstrates the effective purification capability of the PWC hydrogel. The resistance values of water before and after purification were measured using a multimeter in a 5 wt% NaCl solution. The ion concentration of the purified water was significantly lower than that of seawater. Figure S17 shows that the resistance value of the 5 wt% NaCl solution was 15.8 kΩ, while that of the collected condensate water reached 1390 kΩ. This indicates the outstanding desalination performance of the PWC hydrogel. To further evaluate the seawater purification capacity of the PWC hydrogel, four principal ions (Na^+^, Ca^2+^, Mg^2+^, K^+^) from the Bohai Sea were selected for the seawater desalination test. As shown in Figure 8f, the concentrations of these ions decreased by approximately three orders of magnitude, resulting in values much lower than the World Health Organization (WHO) drinking water standards.

## 4. Conclusions

In summary, a high-performance photothermal WC/C composite was successfully synthesized via a one-step molten salt coating method. Through the synergistic effect of WC and carbon, the WC/C composite significantly improved the photothermal conversion efficiency and optical absorption capability compared to pure WC and carbon materials. Specifically, the WC/C composite exhibited a photothermal conversion efficiency of 67.1%, which was 15.4% and 22.5% higher than those of the pure WC and carbon materials, respectively. Moreover, a 3D solar evaporator (PWC) was fabricated using chemical cross-linking and physical freeze–thaw methods. Under 1 kW m^−2^ illumination, the evaporation rate and evaporation efficiency of the hydrogel evaporator with the WC/C nanoparticles achieved 2.99 kg m^−2^ h^−1^ and 90.9% in 5 wt% salt solution. Meanwhile, the PWC hydrogel exhibited long-term salt tolerance performance in a high-salt environment, along with good self-cleaning and organic purification capabilities. The successful preparation of WC/C in our work demonstrates a potential pathway for improving the photothermal conversion capability of traditional carbon materials, which in turn enhances the solar water evaporation efficiency.

## Data Availability

The data that support the findings of this study are available from the corresponding author upon reasonable request. The data supporting this article have been included as part of the Supplementary Materials.

## Supplementary Materials

The following supporting information can be downloaded at: https://www.mdpi.com/article/10.3390/nano16120738/s1, Figure S1: Raman spectra of WC/C with different DCD/AMT mass ratios; Figure S2: XPS spectra of (a) WC/C; (b) O 1s, (c) W 4f, and (d) C 1s; Figure S3: TEM images of a DCD/AMT-2, b DCD/AMT-4, c DCD/AMT-6, and d DCD/AMT-8; Figure S4. EDS image of the WC/C composite; Figure S5: TG, DTA, and DTG curve of WC/C precursor; Figure S6: Comparative UV–Vis–NIR absorption spectra of WC/C with varying DCD/AMT mass ratios; Figure S7: (a) Light intensity-dependent photothermal response curves of WC/C. (b) Linear relationship between T and optical power density; Figures S8–S10: The cooling curves and the corresponding linear relationships between time and ln(θ) for WC/C, C, and WC under 808 nm laser irradiation at 0.19 W cm^−2^; Figure S11: Water vaporization enthalpy of bulk water and water in PVA and PWC; Figure S12: Evaporation rates of pure water, PVA, and PWC under 1 sun illumination; Figure S13. Real-time self-cleaning plots of the PWC hydrogel; Figure S14. Evaporation rate graphs of the PWC evaporator at 5 wt% NaCl concentration for 12 h; Figure S15. Surface images of the PWC evaporator before and after a continuous 12 h salt resistance test (in a 5 wt% NaCl solution); Figure S16: Digital images before and after pressed by 200 g weights of the PWC hydrogel; Figure S17: The resistance values of the water samples before and after desalination with sodium chloride solution; Supplementary Note S1: The calculation mass percentages of WC and carbon in the WC/C composite; Table S1: Mass percentage of WC and carbon in the WC/C composite; Supplementary Note S2: The calculation formula for the photothermal conversion efficiency; Table S2: Efficiency of photothermal conversion (η) of C, WC, and WC/C; Supplementary Note S3: The calculation evaporation rate and solar-vapor conversion efficiency; Table S3: Comparison of evaporation performance between the present work and the published literature. References [56,57,58,59,60,61,62,63,64,65,66,67,68] are cited in the supplementary materials.
