# Supplementary material for: WC/C Composite as an Efficient Photothermal Material for Solar-Driven Seawater Evaporation
Source: Nanomaterials (Basel). 2026 Jun 13;16(12):738. doi: 10.3390/nano16120738 (PMC13306074; doi:10.3390/nano16120738)
Supplement: Supplementary file 1 [file nanomaterials-16-00738-s001.zip › nanomaterials-4361178-supplementary.pdf]

Supplementary Information

WC/C Composite as an Efficient Photothermal Material for Solar-Driven Seawater Evaporation

Shixu Dong [1], Weifeng Li [1,*], Yumei Long [1,2,*]

[1] College of Chemistry, Chemical Engineering and Materials Science, Soochow University, Suzhou 215123, China
[2] The Key Lab of Health Chemistry and Molecular Diagnosis of Suzhou, Soochow University, Suzhou 215123, China
* Correspondence: liweifeng@suda.edu.cn (W.L.); yumeilong@suda.edu.cn (Y.L.)

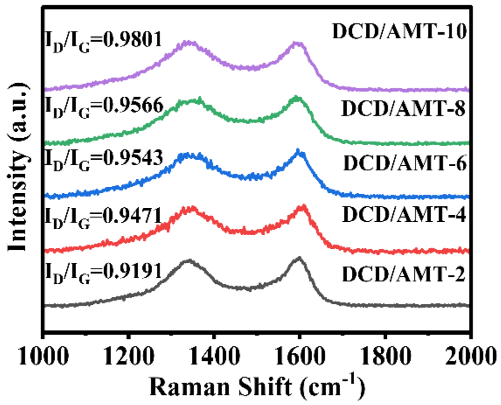

Figure S1. Raman spectra of WC/C with different DCD/AMT mass ratios.

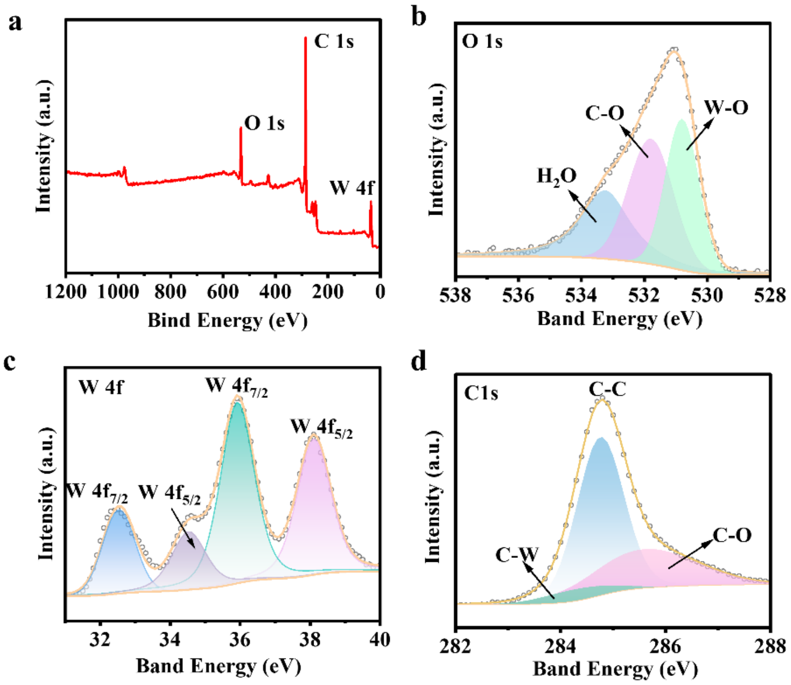

Figure S2. XPS spectra of (**a**) WC/C; (**b**) O 1s, (**c**) W 4f, and (**d**) C 1s.

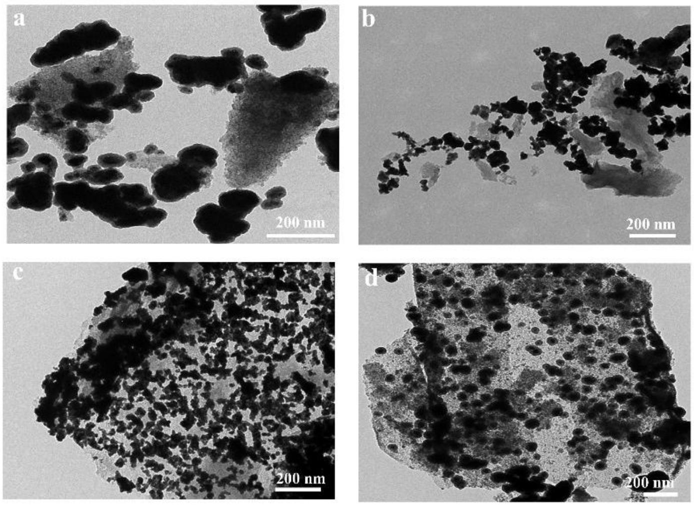

Figure S3. TEM images of **a** DCD/AMT-2, **b** DCD/AMT-4, **c** DCD/AMT-6, and **d** DCD/AMT-8.

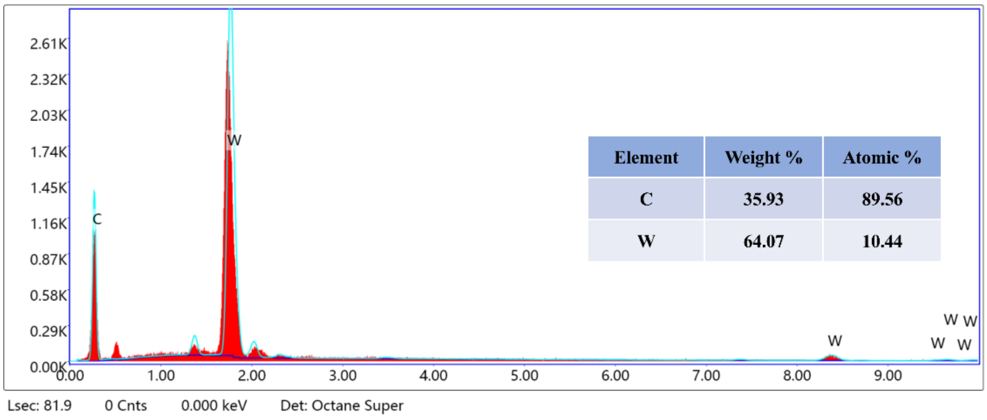

Figure S4. EDS image of the WC/C composite.

Supplementary Note S1: Based on the measured W content (64.07 wt%), the mass fraction of WC in the composite was calculated assuming stoichiometric WC (molar mass = 195.85 g·mol^{-1}, W = 183.84 g·mol^{-1}):

$$\omega_{W/WC} = \frac{M_W}{M_{WC}} = \frac{183.84}{195.85} \times 100\% = 93.87\% \qquad (S1)$$

$$\omega_{C/WC} = \frac{M_C}{M_{WC}} = \frac{12.01}{195.85} \times 100\% = 6.13\% \qquad (S2)$$

Because all W atoms are contained within WC, the mass fraction of WC is simply the stoichiometric ratio:

$$\omega_{WC} = \frac{\omega_W}{M_{\omega_{W/WC}}} = \frac{64.07\%}{93.87\%} \times 100\% = 68.25\% \qquad (S3)$$

The mass fraction of carbon in WC:

$$\omega_{C/WC} = \omega_{wc} \times \omega_{C/WC} = 68.25\% \times 6.13\% = 4.18\% \qquad (S4)$$

The carbon content is then derived by subtracting the carbon originating from WC from the total carbon content:

$$\omega_{Carbon} = \omega_C - \omega_{C/WC} = 35.93\% - 4.18\% = 31.75\% \quad (S5)$$

Thus, the Carbon to WC weight ratio in the sample is approximately**1: 2.15**.

Table S1. Mass percentage of WC and carbon in the WC/C composite.

Material	Weight
C	31.75 %
WC	68.25 %

The mass ratio of WC to carbon is approximately 2.15: 1.

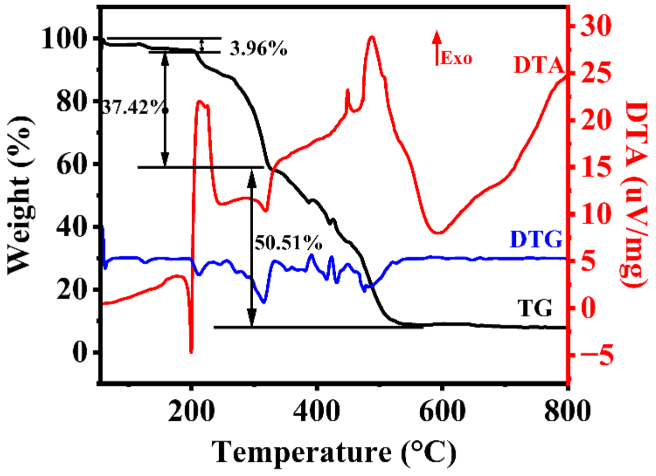

Figure S5. TG, DTA, and DTG curve of WC/C Precursor.

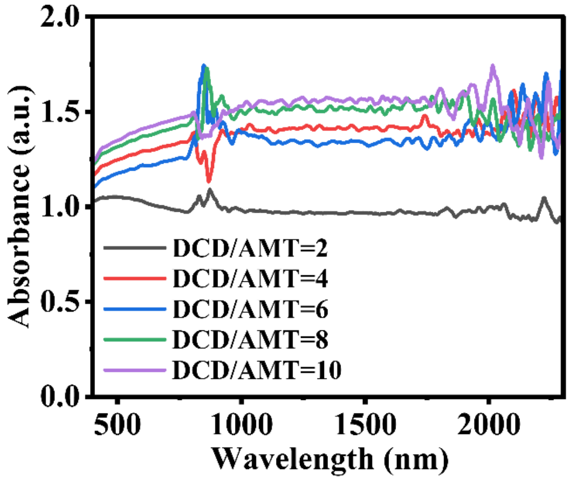

Figure S6. Comparative UV-Vis-NIR absorption spectra of WC/C with varying DCD/AMT mass ratios.

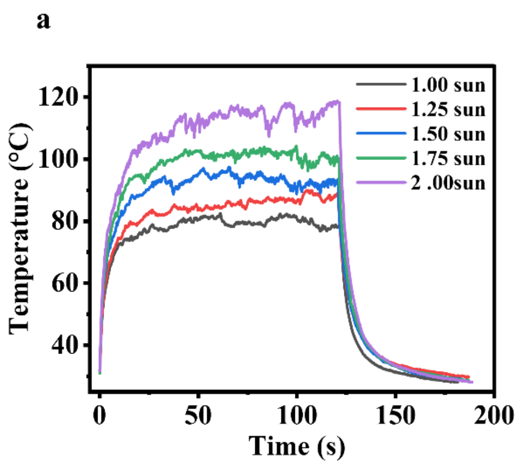

Figure S7. (**a**) Light intensity-dependent photothermal response curves of WC/C; (**b**) Linear relationship between T and optical power density.

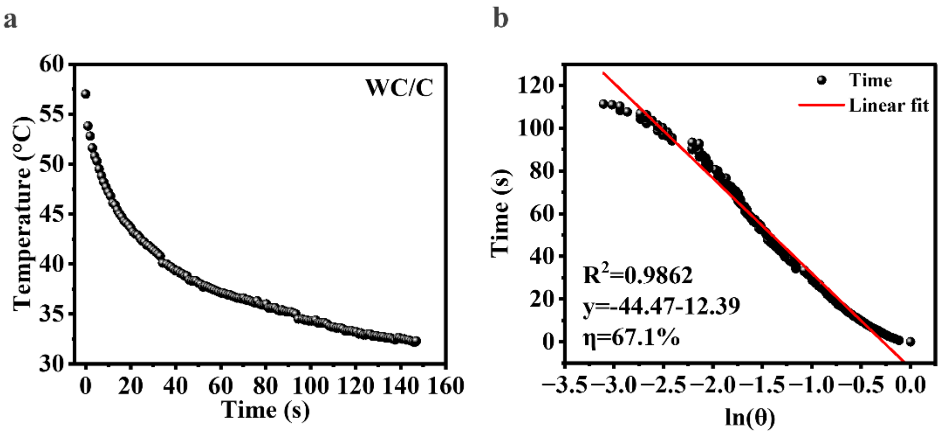

Figure S8. (**a**) The cooling curve of WC/C under 808 nm laser irradiation at 0.19 W cm^{-2}; (**b**) The linear relationship between time and ln(θ).

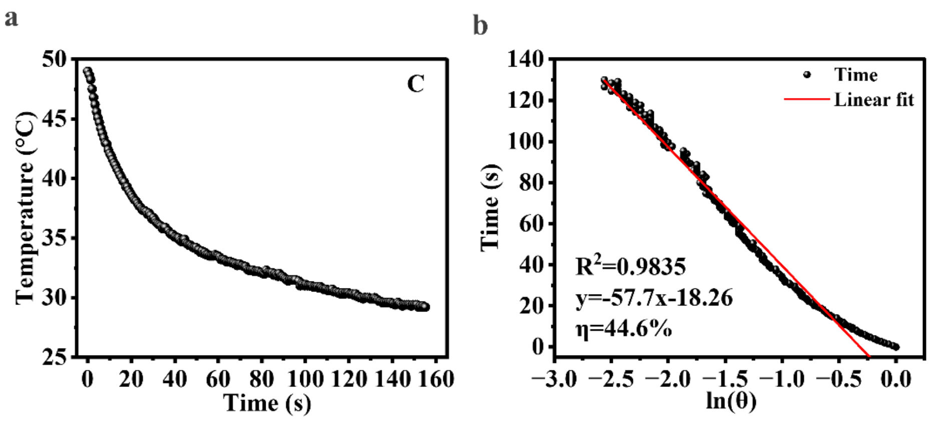

Figure S9. (**a**) The cooling curve of C under 808 nm laser irradiation at 0.19 W cm^{-2}; (**b**) The linear relationship between time and ln(θ).

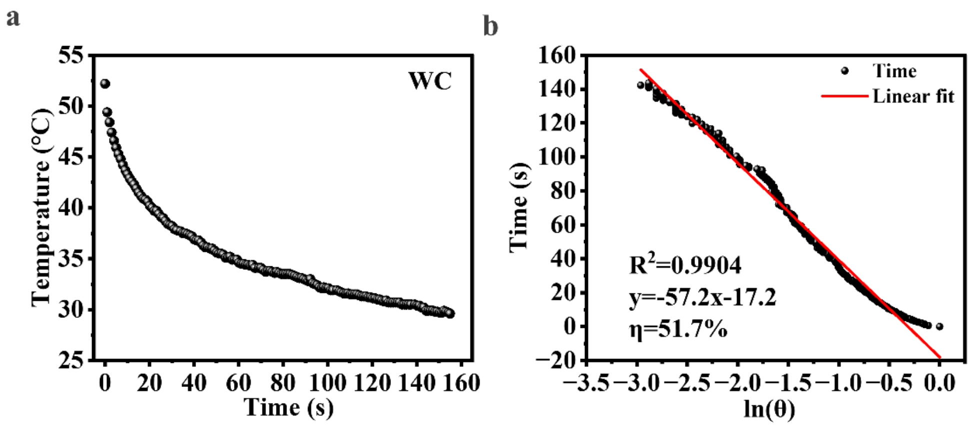

Figure S10. (**a**) The cooling curve of WC under 808 nm laser irradiation at 0.19 W cm^{-2}; (**b**) The linear relationship between time and ln(θ).

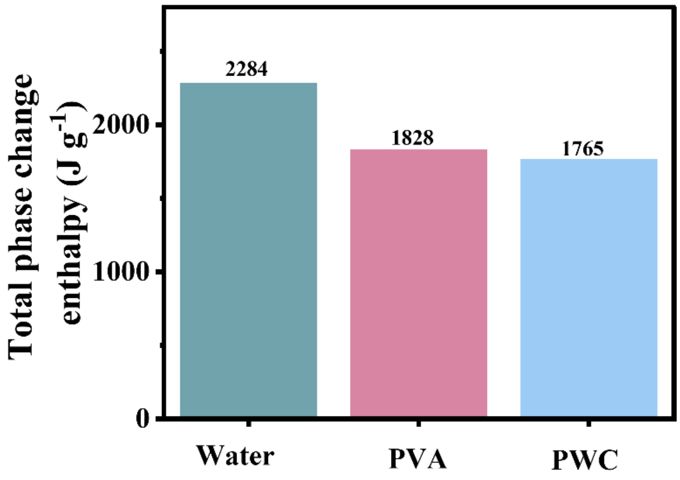

Figure S11. Water vaporization enthalpy of bulk water and water in PVA and PWC.

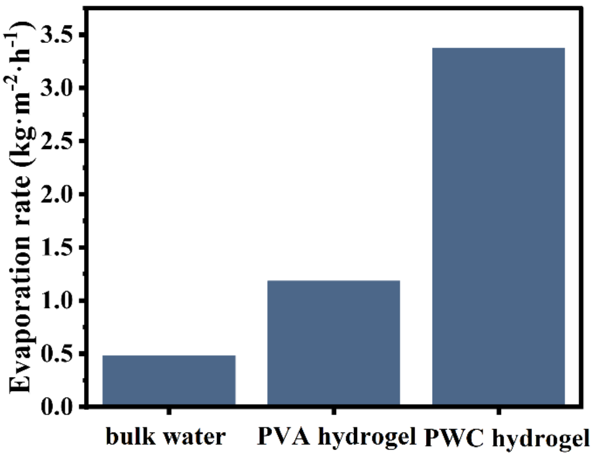

Figure S12. Evaporation rates of pure water, PVA, and PWC hydrogel under 1 kW m^{-2} illumination.

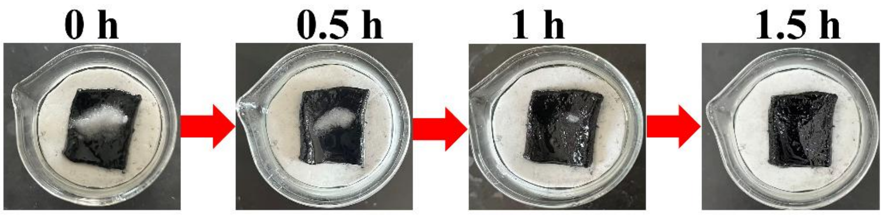

Figure S13. Real-time self-cleaning plots of PWC hydrogel.

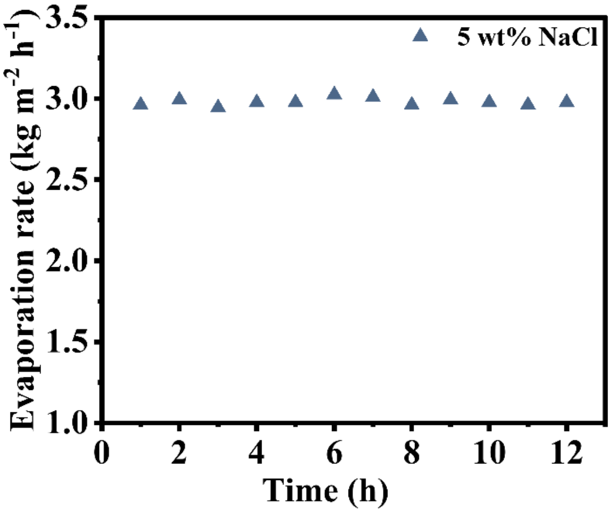

Figure S14. Evaporation rate graphs of the PWC evaporator at 5 wt% NaCl concentration for 12 h.

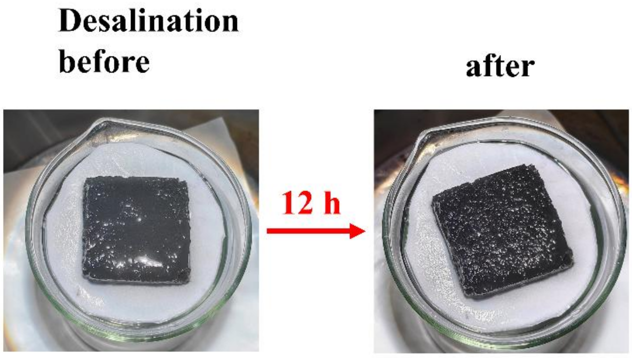

Figure S15. Surface images of PWC evaporator before and after a continuous 12 h salt resistance test (in a 5 wt% NaCl solution).

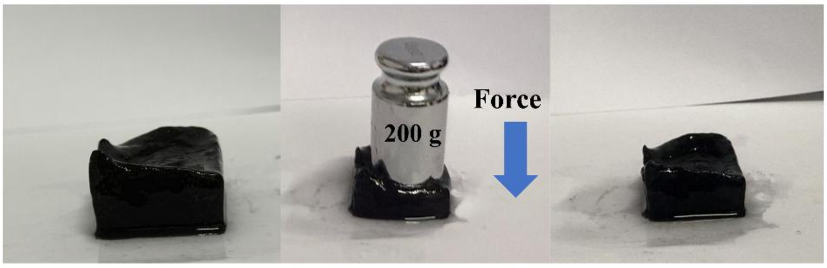

Figure S16. Digital images before and after pressed by 200 g weights of the PWC hydrogel.

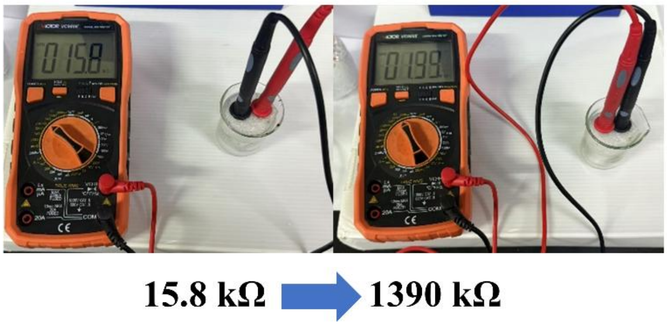

Figure S17. The resistance values of water samples before and after desalination with sodium chloride solution.

Supplementary Note S2: The photothermal properties of the material can also be reflected by the photothermal conversion efficiency. An 808 nm laser was used to irradiate the material placed in the quartz chip slot for 180 seconds, then the laser was turned off, and it was naturally cooled. The calculation formula for the photothermal conversion efficiency is as follows [56]:

Combined with the total energy balance of the system:

$$\sum_i m_i C_{pi} \frac{dT}{dt} = Q_s - Q_{loss} \qquad \text{(S6)}$$

In Equation (1), m_i (0.28 g) and Cp_i (0.8 J/g ℃) respectively represent the mass and specific heat capacity of the components in the system. Since the mass of WC/C in the system is much smaller than that of the quartz plate, the heat capacity of the quartz plate is used for estimation during the calculation process. Q_s is the input energy of the 808 nm laser irradiating the sample, and Q_{loss} is the heat energy diffused to the surrounding environment in the system. When the temperature of the sample irradiated by the laser reaches its maximum, the system is in equilibrium. At this point, the input energy is equal to the heat energy dissipated to the environment, which is expressed by the following formula;

$$Q_s = Q_{loss} = hS\Delta T_{max} \qquad \text{(S7)}$$

Where h is the heat transfer coefficient, S is the area, and ΔT_{max} is the difference between the maximum temperature and the ambient temperature.

The calculation formula for the photothermal conversion efficiency (PEC) of WC/C materials [57]:

$$\eta = \frac{hS\Delta T_{max}}{I\left(1-10^{-A808}\right)} \qquad \text{(S8)}$$

$$\theta = \frac{T - T_{surr}}{T_{max} - T_{surr}} \quad (S9)$$

$$\tau_s = \frac{\sum_i m_i C_{p,i}}{hS} \quad (S10)$$

$$\frac{d\theta}{dt} = \frac{1}{\tau_s}\frac{Q_s}{hS\Delta Tmax} - \frac{\theta}{\tau_s} \quad (S11)$$

Where I is the optical power of the 808 nm laser (0.19 W·cm^{-2}), and A_{808} is the absorbance of the material (WC/C) at 808 nm (1.548). T_{max} is the maximum temperature of the sample (57 °C), and T_{surr} is the ambient temperature (32.4 °C). When the laser is turned off, Q_s in the system is 0.

$$\frac{d\theta}{dt} = -\frac{\theta}{\tau_s} \quad (S12)$$

$$t = -\tau_s ln\theta \quad (S13)$$

Fig. S7 present the cooling profile of WC/C and demonstrate the linear dependence of ln(θ) on time, with θ defined as the driving force temperature. Quantitative analysis yielded a photothermal conversion efficiency of 67.1% for the WC/C composite.

Where *A808* is the ultraviolet absorbance of pure carbon material at 808 nm (1.29), T_{max} is the maximum sample temperature (49 °C), and T_{surr} denotes the surrounding ambient temperature (28.3 °C). The calculated photothermal conversion efficiency of the pure carbon material was determined to be 44.6% (Fig. S8).

Where *A808* is the ultraviolet absorbance of WC material at 808 nm (1.12), T_{max} iis the maximum sample temperature (52.2 °C), and T_{surr} denotes the surrounding ambient temperature (29 °C). The calculated photothermal conversion efficiency of WC was determined to be 51.7% (Fig. S9).

Table S2. Efficiency of Photothermal conversion (η) of C、WC and WC/C

Material	Efficiency of Photothermal conversion (η)
C	44.6%
WC	51.7%
WC/C	67.1%

Supplementary Note S3: The calculation evaporation rate and solar-vapor conversion efficiency: The evaporation performance of the photothermal water collector (PWC) was quantitatively evaluated based on the temporal mass loss of water, with the evaporation rate (v) expressed by the following formula [58]:

$$v = \frac{\Delta m}{S \times \Delta t} \quad (S14)$$

Where Δm is the change in water mass (kg) during the evaporation process, and S indicates the area of the upper surface of the evaporator. t represents the duration of illumination.

Calculation formula for photothermal conversion efficiency η [59]:

$$\eta = \frac{vH_{equ}}{C_{opt}P_0} \quad (S15)$$

where v is the evaporation rate without light conditions, H_{equ} is the equivalent evaporation enthalpy in the hydrogel, C_{opt} is the optical concentration, and P_0 represents the standard solar irradiance (1 kW m^{-2}).

The equivalent enthalpy of evaporation H_{equ} of water can be calculated according to the following formula [60]:

$$\Delta H_{equ} \cdot m_g = \Delta H_{vap} \cdot m_0 \tag{S16}$$

Where ΔH_{vap} and m_0 respectively are the equivalent enthalpy of evaporation and the mass change of water under dark conditions. m_g is the mass change of the evaporator under dark conditions. Among them, the enthalpy of water evaporation is 2450 J g^{-1}. Therefore, the equivalent enthalpy of evaporation in the water gel is calculated to be 1225 J g^{-1}, and the evaporation rate v = 2.67 kg·m^{-2} h^{-1}. Substituting it into the formula of photothermal conversion efficiency η yields 90.9%.

Table S3. Comparison of evaporation performance between the present work and published literature

**Absorbing material**	**Steam generation efficiency (%)**	**Steam generation rate（kg m^{-2} h^{-1}）**	**Refs**
CuS-MoS_2	91.5	1.92	[46]
CNT	88.13	2.16	[61]
CH-TA-Fe^{3+}	73.2	1.83	[62]
PAN@CuS	83.9	2.27	[63]
Au/Ag	82.1	1.31	[64]
rGO-CNTs-$Cs_{0.32}WO_3$	85.9	1.93	[65]
ZrC/GO	94	2.00	[66]
WO_{3-X} NRs	82.5	1.28	[67]
$W_{18}O_{49}$	82	1.15	[68]
**WC/C**	**90.9**	**2.99**	**This work**
